# Outcomes of prevention of mother to child transmission of the human immunodeficiency virus-1 in rural Kenya—a cohort study

**DOI:** 10.1186/s12889-015-2355-4

**Published:** 2015-10-03

**Authors:** Eunice Wambui Nduati, Amin Shaban Hassan, Miguel Garcia Knight, Daniel Muli Muema, Margaret Nassim Jahangir, Shalton Lwambi Mwaringa, Timothy Juma Etyang, Sarah Rowland-Jones, Britta Christina Urban, James Alexander Berkley

**Affiliations:** Kenya Medical Research Institute/Wellcome Trust Research Programme, Centre for Geographic Medicine Research Coast, Kilifi, Kenya; Nuffield Department of Clinical Medicine, University of Oxford, Oxford, UK; Kilifi County Hospital, Kilifi, Kenya; Liverpool School of Tropical Medicine, Liverpool, UK

**Keywords:** HIV, Prevention of mother to child transmission, Vertical transmission, Retention

## Abstract

**Background:**

Success in prevention of mother-to-child transmission (PMTCT) raises the prospect of eliminating pediatric HIV infection. To achieve global elimination, however, strategies are needed to strengthen PMTCT interventions. This study aimed to determine PMTCT outcomes and identify challenges facing its successful implementation in a rural setting in Kenya.

**Methods:**

A retrospective cohort design was used. Routine demographic and clinical data for infants and mothers enrolling for PMTCT care at a rural hospital in Kenya were analysed. Cox and logistic regression were used to determine factors associated with retention and vertical transmission respectively.

**Results:**

Between 2006 and 2012, 1338 infants were enrolled and followed up for PMTCT care with earlier age of enrollment and improved retention observed over time. Mother to child transmission of HIV declined from 19.4 % in 2006 to 8.9 % in 2012 (non-parametric test for trend *p* = 0.024). From 2009 to 2012, enrolling for care after 6 months of age, adjusted Odds Ratio [aOR]: 23.3 [95 % confidence interval (CI): 8.3–65.4], presence of malnutrition ([aOR]: 2.3 [95 % CI: 1.1–5.2]) and lack of maternal use of highly active antiretroviral therapy (HAART) (aOR: 6.5 [95 % CI: 1.4–29.4]) was associated with increased risk of HIV infection. Infant’s older age at enrollment, malnutrition and maternal HAART status, were also associated with drop out from care. Infants who were not actively followed up were more likely to drop out from care (adjusted Hazard Ratio: 6.6 [95 % CI: 2.9–14.6]).

**Discussion:**

We report a temporal increase in the proportion of infants enrolling for PMTCT care before 3 months of age, improved retention in PMTCT and a significant reduction in the proportion of infants enrolled who became HIV-infected, emphasizing the benefits of PMTCT.

**Conclusion:**

A simple set of risk factors at enrollment can identify mother-infant pairs most at risk of infection or drop out for targeted intervention.

## Background

Mother to child transmission (MTCT) remains one of the HIV pandemic’s most important challenges. In the absence of preventive interventions, the risk of acquisition of HIV *in utero* or at birth is 15 to 30 %, increasing to 20 to 45 % with breastfeeding [1, 2]. Prevention of MTCT (PMTCT) strategies can reduce HIV vertical transmission to less than 1 % in developed countries [1, 3] but despite positive strides, less success is being achieved in middle- and low-income countries [4].

There has been a call for “elimination” of pediatric HIV [5], defined as 90 % reduction of new infant infections and a decrease of MTCT to <5 %. However, global coverage of PMTCT services remains below what is required to meet this goal [6]. In sub-Saharan Africa, PMTCT coverage is highly variable, with only 5 countries attaining >80 % coverage [7, 8]. In addition, reports of PMTCT coverage usually reflect enrollment and initiation of PMTCT interventions, rather than the completion of a multifaceted PMTCT cascade.

A diverse range of maternal, cultural and economic challenges hinder the success of PMTCT in developing countries [9, 10]. A recent review of data from low and middle-income regions estimated PMTCT programme losses of 49 % amongst HIV-infected pregnant women between registration at the antenatal clinic and delivery, loss of about 34 % of HIV exposed infants by 3 months of age and loss of 45 % after HIV testing [9]. Malnutrition offers additional challenges: growth impairment is reported in infants born to HIV-infected mothers, including HIV-exposed but uninfected infants [11, 12]. Poor nutritional status in HIV-infected pregnant women may additionally impair immunity and weaken epithelial integrity, which are associated with vertical transmission of HIV [13]. Furthermore, acute maternal HIV infection late in pregnancy or during breastfeeding may go unnoticed and it is associated with very high risk of MTCT [14]. Vertical infection is estimated at 27 % during acute infection [15, 16] compared to 9 to 16 % in chronically infected breastfeeding women in the absence of therapy [17, 18].

If elimination of MTCT is to be achieved, then strategies need to address the whole cascade of interventions. In this study we aimed to describe retention in PMTCT care up to 18 months of age, risk factors for non-retention and the rate of MTCT of HIV infection amongst HIV-exposed infants at a rural hospital in Kenya.

## Methods

### Study site

The study was conducted at the Comprehensive Care and Research Clinic (CCRC), Kilifi County Hospital (KCH), prior to a national recommendation in 2012 for integration of PMTCT services with Mother to Child Health (MCH) services. At the time of the study, HIV infected mothers delivering at the maternity department, KCH or peripheral clinics were referred to the CCRC for continued PMTCT care as per the Kenyan guidelines [19]. In summary, the guidelines recommended that all pregnant women should be tested for HIV during their first antenatal clinic visit and that a repeat test should be offered to initially HIV negative women during the third trimester. Mothers would be placed on life-long highly active antiretroviral therapy (HAART) if their CD4 count was less than 350 cells/mm^3^ but if higher, on prophylactic antiretroviral therapy azidothymidine (AZT) from 14 weeks of pregnancy (or at first contact with antenatal services, if later) and AZT prophylaxis continued through labour and 1 week after delivery. HIV exposed infants born to mothers not on HAART were prescribed nevirapine prophylaxis at birth to be continued until 1 week after complete cessation of breastfeeding while those with mothers on HAART, nevirapine prophylaxis stopped at 6 weeks of life. Infants aged less than 18 months were tested for HIV by PCR at 6 weeks after birth or at the earliest opportunity, subsequently an antibody test at 9 months (if previously PCR negative) and 18 months was performed. Infants with confirmed HIV infection at any of these test points were immediately put on HAART. All HIV-exposed infants were given prophylactic cotrimoxazole during the first 18 months of life and those testing HIV positive at any of the testing time points continued on life-long cotrimoxazole. HAART and cotrimoxazole were supplied at monthly visits.

### Study population

We examined data from mother-infant pairs enrolling for PMTCT care between 2009 and 2012. To assess PMTCT completion we included infants who were under 18 months old on 1^st^ January 2009, but over 18 months on 31^st^ December 2013, which ensured that infants had sufficient time to complete the 18 months follow up. In 2011, a sub-set of infants were recruited into a study assessing immune responses in HIV-exposed infants (outcomes reported elsewhere). Infants recruited in the immune response study received similar PMTCT care to other infants at the clinic, but had study-related 3-monthly follow-up visits, scheduled to coincide with one of the infant’s routine PMTCT monthly visits. Defaulters in the immune response study initially received two telephone calls then a trained fieldworker was dispatched to encourage the caregiver in bringing in the infant for care. Transport costs for study related visits were reimbursed.

To examine numbers enrolled and changes in the proportion of exposed infants who became HIV-infected over time, we added data from our previous report on early infant diagnosis of HIV infection from 2006 to 2008 [20].

### Study design

In a retrospective cohort design, routinely collected data from all eligible infants were considered in the analyses. Where possible, infant data were linked to maternal data at delivery.

### Sources of data and outcome definitions

Infants’ demographic and clinical data including gender, residence and distance in kilometers to the hospital, date of birth, date of enrollment into care, anthropometry, and HAART use and prophylaxis were systematically captured at each visit. Similarly, mothers’ demographic and clinical data including age, education status, marital status, body mass index (BMI), with a BMI of <18.5 considered as malnutrition, CD4 T-cell count and HAART use were routinely captured.

In infants, malnutrition was defined as wasting (weight-for-height z-score [WHZ < −2.0]), stunting (height-for-age Z score [HAZ < −2.0]) and underweight (weight-for-age Z score [WAZ < −2.0]) [21].

Retention in PMTCT was defined as infants remaining in follow-up up to the recommended 18 months of age. MTCT of HIV-1 was defined as infants who either tested positive by polymerase chain reaction (PCR) at 6 weeks (or at first contact) or by antibody tests at 9 or 18 months.

### Sample size estimation

A *post hoc* sample size estimation was done as analyses in this study were performed on routinely collected data. We previously reported retention of 35 % among HIV-exposed infants enrolled for care and followed up to 18 months of age [20]. Assuming similar retention rates, the likelihood of 600 HIV-exposed infants being in follow-up to 18 months of age would be estimated with a precision of +/−4 % at 95 % confidence levels. This number also allows the risk of MTCT of HIV infection to be described with a precision of about +/−2 %, assuming an estimated MTCT incidence of 6.5 % [22] and the prevalence of malnutrition with a precision of about +/−3 % assuming an estimated prevalence of 15 % [23].

### Data analysis

Continuous data were presented using medians and interquartile ranges (IQR) and categorical data using frequencies and percentages. Variables with more than 10 % missing data were considered, with missing recorded as a separate category and included in the analyses as such. Logistic regression models were used to describe independent correlates of MTCT of HIV-1 infection. Odds ratios (OR), 95 % confidence intervals (CI) and Likelihood Ratio Test (LRT) *p*-values were presented. Cox proportional hazards regression models were used to assess independent predictors of time to loss-to-follow up. Hazard Ratios (HR), 95 % CI and LRT *p*-values were presented. We were unable to link a quarter of the HIV-exposed infants to their mothers’ data. For this reason, regression models for each of the outcomes as described above were applied separately and independently for infants and mothers. All variables were included in the multivariable models to adjust for any negative or positive residual confounding effects. All analyses were carried out using STATA v12.0 (StataCorp, College Station, Texas, USA).

### Ethical considerations

Data analysed had been routinely collected at the clinic and personal identifiers dropped. For the subset of mother-infant pairs under active follow-up, written informed consent was obtained from the mother. Ethical permission for this study was granted by the National Ethics and Review Committee, Kenya Medical Research Institute with reference number ERC 2085.

## Results

### Baseline characteristics at enrollment into care

**Fig. 1:**
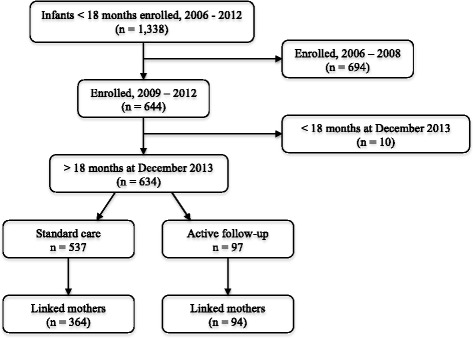
Enrollment for PMTCT care at a rural HIV clinic in Kenya. Infants enrolled between 2009 and 2012, and older than 18 months as at 31 December 2013 (*n* = 634), were matched to their mothers (*n* = 458) and used in the main analysis. Infants enrolled since 2006 were included in the description of changes in enrollment and in HIV transmission over the years (*n* = 1338). PMTCT; Prevention of Mother to Child HIV-1 Transmission

Between 2006 and 2012, 1338 infants under 18 months were enrolled for HIV care at the clinic. Of these 634 were enrolled between 2009 through 2012, were aged 18 months and above by December 2013 and included in the main analysis (Fig. 1). From November 2011, 97 of the 634 infants were actively followed-up in a study of immune responses. Overall, we were able to link 458/634 [72 %] of the infants to their mothers’ data.

**Fig. 2:**
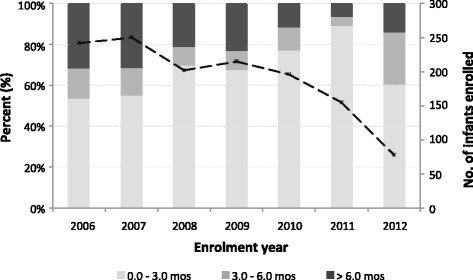
Temporal changes in infant enrollment for PMTCT care at a rural HIV clinic in Kenya. Dashed line: infants enrolled each year. Light grey, mid-grey and dark-grey bars represent age at enrollment: enrolled before 3 months, between 3 to 6 months and after 6 months of age respectively. The total number of infants enrolled (*n* = 1338). PMTCT; Prevention of Mother to Child HIV-1 Transmission

The number of infants enrolling at the clinic for PMTCT systematically dropped over the years (Fig. 2), a trend similar to enrollment for adult HIV care at the same clinic (*data not shown*). The number of infants enrolling for PMTCT within the first 3 months of life significantly increased over the study period (non-parametric test for trend, *p* < 0.001). Overall, 475 [74.9 %] infants enrolled before 3 months of age, and the median age of enrollment of infants into care was 0.8 (IQR, 0.3 to 3.0) months (Table 1). The majority of the infants were female (*n* = 366 [57.7 %]), and 40 % (*n* = 246) lived within 5 km of the hospital.

**Table 1:** Characteristics of HIV-exposed infants and their HIV-1 infected mothers enrolled for care at a rural HIV clinic in Kenya (*N* = 634)

		Standard Care	Active follow up	Overall Care
Infants Characteristics		*N* = 537 [%]	*N* = 97 [%]	*N* = 634 [%]
Gender	Male	224 [41.7]	44 [45.4]	268 [42.3]
	Female	313 [58.3]	53 [54.6]	366 [57.7]
Age at intake (months)	Median (IQR)	0.9 (0.3–3.5)	0.6 (0.3–1.5)	0.8 (0.3–3.0)
Age group at enrollment (months)	0–3	394 [73.4]	81 [83.5]	475 [74.9]
	3–6	52 [9.7]	13 [13.4]	65 [10.3]
	>6	91 [17.9]	3 [3.1]	94 [14.8]
Hospital distance (kilometers)	Median (IQR)	7.4 (2.2–13.4)	7.8 (2.2–17.7)	7.4 (2.2–13.4)
Hospital distance categories (kilometers)	0–5	206 [38.4]	40 [41.2]	246 [38.8]
	5–10	136 [25.3]	22 [22.7]	158 [24.9]
	>10	152 [28.3]	34 [35.1]	186 [29.3]
	Missing	43 [8.0]	1 [1.0]	44 [6.9]
Mothers Characteristics (at infant’s birth)		*N* = 364 [%]	*N* = 94 [%]	*N* = 458 [%]
Age (years)	Median (IQR)	28.1 (23.3–33.5)	28.9 (24.8–34.9)	28.4 (23.7–33.8)
Age group (years)	<25	120 [33.0]	24 [25.5]	144 [31.4]
	25–35	177 [48.6]	47 [50.0]	224 [48.9]
	>35	67 [18.4]	23 [24.5]	90 [19.7]
Education status	No education	133 [36.5]	30 [31.9]	163 [35.6]
	Primary	165 [45.3]	41 [43.6]	206 [45.0]
	>Secondary	39 [10.7]	11 [11.7]	50 [10.9]
	Missing	27 [7.4]	12 [12.8]	39 [8.5]
BMI	Median (IQR)	21.0 (19.2–23.4)	21.4 (19.1–23.2)	21.0 (19.2–23.3)
BMI categories	<18.5	56 [15.4]	15 [16.0]	71 [15.5]
	>18.5	296 [81.3]	77 [81.9]	373 [81.4]
	Missing	12 [3.3]	2 [2.1]	14 [3.1]
CD4 count	Median (IQR)	409 (264–549)	420 (300–640)	410 (279–571)
CD4 count categories	<350	85 [23.4]	25 [26.6]	110 [24.0]
	>350	121 [33.2]	40 [42.6]	161 (35.2)
	Missing	158 [43.4]	29 [30.9]	187 (40.8)
Duration on HAART (months)	Median (IQR)	20.6 (2.9–36.9)	28.1 (7.1–44.6)	24.9 (3.4–39.3)
Duration on HAART categories (months)	Not on HAART^a^	229 [62.9]	50 [53.2]	279 [60.9]
	0–24	71 [19.5]	17 [18.1]	88 [19.2]
	>24	64 [17.6]	27 [28.7]	91 [19.8]

Infants missing mothers data (*n* = 176 [28 %]); BMI: body mass index, HAART: Highly active antiretroviral therapy, *IQR* interquartile range

^a^mothers not on HAART received prophylactic antiretroviral therapy azidothymidine (AZT) during, through and after delivery, as per the Kenyan guidelines at the time of the study, ref [19] if they had attended antenatal clinic

The median maternal age at delivery was 28 (IQR, 24 to 34) years. Most mothers had primary education or less (*n* = 369 [80.6 %]) and were not malnourished at the time of delivery (BMI >18.5, *n* = 373 [81.4 %]). Their median CD4 count at delivery was 410 (IQR, 279 to 571). The majority of mothers were not on long-term HAART (*n* = 279 [60.9 %]), with only a small proportion [19.8 %] on continuous HAART for more than 2 years prior to delivery.

Ninety-seven infants were enrolled into the immune response study. Their baseline characteristics at enrollment did not differ from those of infants under standard care (Table 1).

### Malnutrition at enrollment into care

Of the 634 exposed infants enrolled for HIV care during the study period, 560 (88.3 %) had baseline weight, age and height data available. A total of 285 infants (53.1 %) exhibited at least one of the malnutrition syndromes (wasting, underweight or stunting) and 26 (4.8 %) had a combination of all syndromes. The overall prevalence of wasting, underweight and stunting was 14.0, 24.1 and 41.5 % respectively (Table 2) and worsened with older age at enrollment (correlation coefficients [95 % CI]: −0.096 ([95 % CI: −0.143 to −0.049], *p* < 0.001); −0.099 ([95 % CI: −0.136 to −0.062], *p* < 0.001) and −0.060 ([95 % CI: −0.105 to −0.016], *p* = 0.008), respectively. Neither the infant’s gender, residential area nor were maternal characteristics associated with the infant’s nutritional status at enrollment.

**Table 2:** Distribution of nutritional status amongst HIV-exposed infants at enrollment for PMTCT care at a rural HIV clinic in Kenya (*N* = 634)

		^a^Wasting, *n* = 479 [%]	^b^Underweight *n* = 526 [%]	^c^Stunting *n* = 528 [%]	^d^Any *n* = 537 [%]	^e^All *n* = 539 [%]
Infants Characteristics		67/479 [14.0]^f^	135/526 [24.1]^f^	219/528 [41.5]^f^	285/537 [53.1]^f^	26/539 [4.8]^f^
Gender	Male	34/212 [16.0]	67/241 [27.8]	100/229 [43.7]	128/233 [54.9]	15/232 [6.5]
	Female	33/267 [12.4]	68/319 [21.3]	119/299 [39.8]	157/304 [51.6]	11/307 [3.6]
Age group (months)	0–3	38/352 [10.8]	80/425 [18.8]	157/403 [39.0]	202/407 [49.6]	9/407 [2.2]
	3–6	9/51 [17.7]	19/56 [33.9]	23/48 [47.9]	33/52 [63.5]	4/53 [7.6]
	>6	20/76 [26.3]	36/79 [45.6]	39/77 [50.7]	50/78 [64.1]	13/79 [16.5]
Hospital distance (kilometers)	0–5	23/184 [12.5]	49/221 [22.2]	85/208 [40.9]	110/209 [52.6]	8/215 [3.7]
	5–10	15/125 [12.0]	41/144 [28.5]	56/135 [41.5]	75/141 [53.2]	5/134 [3.7]
	>10	23/146 [15.8]	39/168 [23.2]	69/161 [42.9]	86/161 [53.4]	10/164 [6.1]
Cohort	Standard Care	58/395 [14.7]	127/465 [27.3]	185/436 [42.4]	243/445 [54.6]	23/443 [5.2]
	Active follow up	9/84 [10.7]	8/95 [8.4]	34/92 [37.0]	42/92 [45.7]	3/96 [3.1]
Mothers’ Characteristics						
Age at infant’s birth (years)	<25	18/116 [15.5]	32/141 [22.7]	52/134 [38.8]	70/135 [51.9]	5/134 [3.7]
	25–35	24/191 [12.6]	39/220 [17.7]	81/209 [38.8]	106/212 [50.0]	8/214 [3.7]
	>35	6/74 [8.1]	12/87 [13.8]	32/82 [39.0]	40/85 [47.1]	1/82 [1.2]
Education status	No education	15/129 [11.6]	34/159 [21.4]	64/148 [43.2]	81/150 [54.0]	6/149 [4.0]
	Primary	24/176 [13.6]	38/202 [18.8]	69/194 [35.6]	95/197 [48.2]	6/196 [3.1]
	>Secondary	6/44 [13.6]	6/50 [12.0]	17/49 [34.7]	23/50 [46.0]	1/48 [2.1]
BMI at infants birth	<18.5	9/55 [16.4]	17/69 [24.6]	29/66 [43.9]	38/67 [56.7]	3/63 [4.8]
	>18.5	37/315 [11.8]	63/366 [17.2]	133/346 [38.4]	173/353 [49.0]	11/354 [3.1]
CD4 count at infants birth	<350	13/88 [14.8]	21/107 [19.6]	40/100 [40.0]	55/104 [52.9]	4/104 [3.9]
	>350	15/136 [11.0]	23/158 [14.6]	55/154 [35.7]	68/153 [44.4]	4/152 [2.6]
	missing	39/255 [15.3]	91/295 [30.9]	124/274 [45.3]	162/280 [57.9]	18/283 [6.4]
Duration on HAART (months)	Not on HAART^g^	34/238 [14.3]	56/273 [20.5]	101/255 [39.6]	135/262 [51.5]	11/265 [4.2]
	0–24	6/70 [8.6]	11/86 [12.8]	28/80 [35.0]	34/81 [42.0]	2/81 [2.5]
	>24	8/73 [11.0]	16/89 [18.0]	36/90 [40.0]	47/89 [52.8]	1/84 [1.2]

*BMI* body mass index; *HAART* Highly active antiretroviral therapy; % percentages; *PMTCT* Prevention of mother-to-child transmission

^a^Wasting: weight for height Z scores < −2.0

^b^Underweight: weight for age Z score < −2.0

^c^Stunting: height for age Z score < −2.0

^d^Any: infant with any of the three malnutrition syndromes

^e^All: infant with all three malnutrition syndromes

^f^Different denominators arising from exclusion of missing data and extreme outliers after derivation of the Z-scores as outlined in the WHO reference data sets ref [21]

^g^mothers not on HAART received prophylactic antiretroviral therapy azidothymidine (AZT) during, through and after delivery, as per the Kenyan guidelines at the time of the study, ref [19] if they had attended antenatal clinic

### Retention in care over the recommended PMTCT 18-month period

Overall, 634 infants were followed up and contributed a total of 6577 person months of observation (pmo). Of these, 247 (39.0 % [95 % CI, 35.1 to 42.9]) were lost to follow-up before 18 months of age: incident rate 3.76 (95 % CI, 3.3 to 4.2)/100 pmo. Sixty-one (9.6 %) of the nfants were enrolled but did not return for any follow-up care, and by 9 months of age, 158 (26.4 %) had dropped out.

The infant’s age, calendar year at enrollment, nutritional status and active follow-up, were independently associated with non-retention in care. Infants enrolled at >6 months of age were almost twice as likely to drop out (Table 3). Infants enrolled in later calendar years had better retention in care (*p* = 0.003). Infants with malnutrition syndromes at enrollment were more likely to drop out compared to those without malnutrition. Infants not on active follow-up were more likely to drop out of care, compared to those receiving active follow-up (HR [95 % CI] 6.6 [2.9 to 14.6], *p* < 0.001).

**Table 3:** Predictors of retention in care amongst HIV-exposed infants enrolled for PMTCT (*N* = 634)

		Non-retention	Cox Univariable analysis		Cox Multivariable analysis	
Infants Characteristics		d/y [rate/100 pmo]^a^	Crude HR [95 % CI]	LRT *p*-value^b^	Adjusted HR [95 % CI]^b^	LRT *p*-value^b^
Gender	Male	105/28.5 [3.7]	Reference		Reference	
	Female	142/37.3 [3.8]	1.0 [0.8–1.3]	0.810	1.1 [0.8–1.4]	0.662
Age group at intake (months)	0–3	179/56.9 [3.1]	Reference		Reference	
	3–6	30/5.1 [5.8]	1.8 [1.2–2.7]		2.1 [1.2–3.4]	
	>6	38/3.8 [10.1]	2.6 [1.8–3.8]	<0.001	1.8 [1.1–3.0]	0.005
Year of intake	2009	113/19.2 [5.9]	Reference		Reference	
	2010	69/20.2 [3.4]	0.6 [0.4–0.8]		0.5 [0.4–0.8]	
	2011	37/19.7 [1.9]	0.3 [0.2–0.5]		0.5 [0.3–0.8]	
	2012	28/6.7 [4.2]	0.7 [0.5–1.1]	<0.001	0.7 [0.4–1.4]	0.003
Any malnutrition at enrollment (WHZ/WAZ/HAZ <−2.0)	No	78/31.3 [2.5]	Reference		Reference	
	Yes	111/29.7 [3.7]	1.5 [1.1–2.0]	0.008	1.5 [1.1–2.0]	0.018
All malnutrition at enrollment (WHZ & WAZ & HAZ < −2.0)	No	175/59.6 [2.9]	Reference		Reference	
	Yes	13/1.2[10.7]	3.4 [1.9–6.0]	<0.001	2.5 [1.3–4.7]	0.014
Hospital distance (kilometers)	0–5	95/27.1 [3.5]	Reference		Reference	
	5–10	57/17.2 [3.3]	0.9 [0.7–1.3]		0.9[0.6–1.4]	
	>10	70/18.6 [3.8]	1.1 [0.8–1.4]	0.818	1.1[0.8–1.6]	0.624
Cohort	Active follow up	7/14.7 [0.5]	Reference		Reference	
	Standard Care	240/51.1 [4.7]	9.2 [4.3–19.5]	<0.001	6.6 [2.9–14.6]	<0.001
Mothers Characteristics						
Age at infants birth (years)	<25	53/17.3 [3.1]	Reference		Reference	
	25–35	81/28.0 [2.9]	1.0 [0.7–1.3]		1.2 [0.8–1.8]	
	>35	24/11.4 [2.1]	0.7 [0.4–1.1]	0.247	0.9 [0.5–1.6]	0.379
Education status	No education	52/20.6 [2.5]	Reference		Reference	
	Primary	73/25.4 [2.9]	1.2 [0.8–1.7]		1.2 [0.8–1.7]	
	>Secondary	17/6.4 [2.7]	1.1 [0.6–1.8]	0.712	1.0 [0.5–1.8]	0.677
BMI at infants birth	<18.5	33/8.5 [3.9]	1.6 [1.1–2.4]		1.6 [1.1–2.5]	
	>18.5	114/47.4 [2.4]	Reference	0.016	Reference	0.036
CD4 count at infants birth	>350	54/21.6 [2.5]	Reference		Reference	
	<350	39/15.2 [2.6]	1.0 [0.7–1.6]		1.2 [0.7–1.8]	
	Missing	65/19.9 [3.3]	1.3 [0.9–1.9]	0.254	1.2 [0.8–1.8]	0.685
Duration on HAART (months)	Not on HAART^c^	112/31.1 [3.6]	2.7 [1.6–4.5]		2.7 [1.5–4.7]	
	0–24	28/12.2 [2.3]	1.7 [0.9–3.1]		1.9 [1.0–3.7]	
	>24	18/13.3 [1.4]	Reference	<0.001	Reference	<0.001

*HR* hazard ratios; *CI* confidence intervals; *LRT* Likelihood Ratio Tests; *WHZ* weight for height, *WAZ* weight for age, *HAZ* height for age z score; *BMI* body mass index; *HAART* highly active antiretroviral therapy; *PMTCT* prevention of mother-to-child transmission

^a^d represents the number of events; y represents person months; PMO: time to loss to follow up is presented as the rate per 100 person months observed [rate/100pmo]

^b^Likelihood of an infant enrolled for Prevention of mother to child transmission of HIV dropping out of care before 18 months of life

^c^mothers not on HAART received prophylactic antiretroviral therapy azidothymidine (AZT) during, through and after delivery, as per the Kenyan guidelines at the time of the study, ref [19] if they had attended antenatal clinic

Infants born to mothers with malnutrition at the time of the infant’s birth had a higher rate of drop out from care compared to those born to mothers without malnutrition (HR [95 % CI] 1.6 [1.1 to 2.5], *p* = 0.036). Mothers not on HAART for more than 2 years prior to the infant’s delivery were almost three-fold more likely to drop out of PMTCT care compared to mothers on long-term HAART (Table 3).

### Correlates of MTCT of HIV-1 infection amongst infants enrolled for PMTCT care

**Fig. 3:**
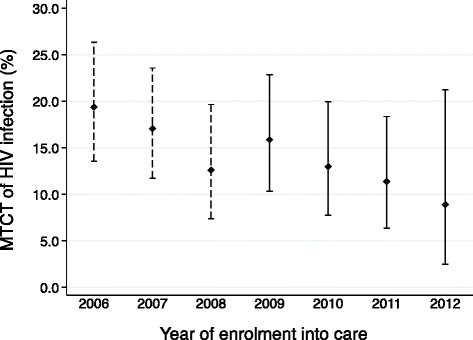
Prevalence of HIV vertical transmission among infants enrolled for PMTCT care at a rural HIV clinic in Kenya Dotted lines: prevalence during previous study, solid lines: prevalence during current study. Non-parametric test for trend, *p* = 0.024, confidence interval (CI) indicated. Total number of infants (*n* = 1338). PMTCT; Prevention of Mother to Child HIV-1 Transmission, MTCT; Mother to child Transmission of HIV

The proportion of enrolled infants who became HIV-infected declined from 19.4 % in 2006 to 8.9 % in 2012 [non-parametric test for trend, *p* = 0.024] (Fig. 3). Of the 634 infants within the study period (2009 to 2013), HIV test results were available for 444 (70.0 %) infants. Overall, 57 infants became infected before age 18 months, suggesting an overall MTCT risk during follow up of 12.8 % (95 % CI, 10.4–16.9) [Table 4]. More than half of the infants who enrolled into care after 6 months of age became HIV infected before age 18 months.

**Table 4:** Correlates of mother to child transmission of HIV-1 infection amongst HIV-1 exposed infants enrolled for care (*N* = 634)

		HIV transmission [%]	Logistic Univariable analysis		Logistic Multivariable analysis	
Infants Characteristics		*n* = 57/444^a^ [12.8]	Crude OR [95 % CI]	LRT *p*-value^b^	Adjusted OR [95 % CI]	LRT *p*-value^b^
Gender	Male	21/195 [10.8]	Reference		Reference	
	Female	36/249 [14.5]	1.4 [0.8–2.5]	0.246	1.4 [0.6–2.9]	0.431
Age group (months)	0–3	28/367 [7.6]	Reference		Reference	
	3–6	10/42 [23.8]	3.8 [1.7–8.5]		3.9 [1.3–12.2]	
	>6	19/35 [54.3]	14.4 [6.7–31.0]	<0.001	23.3 [8.3–65.4]	<0.001
Year of intake	2009	23/145 [15.9]	Reference		Reference	
	2010	16/131 [12.2]	0.7[0.4–1.5]		1.3 [0.5–3.6]	
	2011	14/123 [11.4]	0.7[0.3–1.4]		2.0 [0.6–6.3]	
	2012	4/45 [8.9]	0.5 [0.2–1.6]	0.553	0.6 [0.1–3.5]	0.480
Any malnutrition at intake (WHZ/WAZ/HAZ < −2.0)	No	14/201 [7.0]	Reference		Reference	
	Yes	35/215 [16.3]	2.6 [1.4–5.0]	0.003	2.3 [1.0–5.2]	0.038
All malnutrition at intake (WHZ & WAZ & HAZ < −2.0)	No	44/397 [11.1]	Reference		Reference	
	Yes	8/16 [50.0]	8.0 [2.9–22.4]	<0.001	2.6 [0.6–10.8]	0.203
Hospital distance categories (kilometers)	0–5	20/180 [11.1]	Reference		Reference	
	5–10	9/117 [7.7]	0.7 [0.3–1.5]		0.6 [0.2–1.8]	
	>10	24/130 [18.5]	1.8 [1.0–3.4]	0.032	2.2 [0.9–5.2]	0.043
Cohort	Active follow up	7/96 [7.3]	Reference		Reference	
	Standard Care	50/348 [14.4]	2.1 [0.9–4.9]	0.052	0.6 [0.2–1.8]	0.329
Mothers Characteristics						
Age at infants birth (years)	<25	12/114 [10.5]	Reference		Reference	
	25–35	14/192 [7.3]	0.7 [0.3–1.5]		0.9 [0.4–2.1]	
	>35	8/74 [10.8]	1.0 [0.4–2.7]	0.518	1.7 [0.6–5.1]	0.435
Education status	No education	11/133 [8.3]	Reference		Reference	
	Primary	16/175 [9.1]	1.1 [0.5–2.5]		1.1 [0.5–2.6]	
	>Secondary	2/41 [4.9]	0.6 [0.1–2.7]	0.641	0.6 [0.1–2.9]	0.710
BMI at infants birth	<18.5	6/59 [10.2]	1.2 [0.5–2.9]		1.3 [0.4–3.6]	
	>18.5	28/315 [8.9]	Reference	0.757	Reference	0.660
CD4 count at infants birth	>350	9/144 [6.3]	Reference		Reference	
	<350	7/98 [7.1]	1.2 [0.4–3.2]		1.5 [0.5–4.6]	0.215
	Missing	18/138 [13.0]	2.3 [1.0–5.2]	0.113	2.2 [0.9–5.4]	
Duration on HAART (months)	Not on HAART^c^	28/220 [12.7]	3.9 [1.1–13.2]		6.5 [1.4–29.4]	
	0–24	3/77 [3.9]	1.1 [0.2–5.5]		1.8 [0.3–11.2]	
	>24	3/83 [3.6]	Reference	0.006	Reference	0.004

% percentage; *OR* Odds ratio; *CI* confidence intervals; *LRT* Likelihood Ratio Tests, *p*-values presented; *whz* weight for height; *waz* weight for age; *haz* height for age z scores; *BMI* body mass index; *HAART* highly active antiretroviral therapy

^a^number of infants for whom HIV test results were available

^b^Likelihood of infants enrolled for prevention of mother to child transmission of HIV care being vertically infected

^c^mothers not on HAART received prophylactic antiretroviral therapy azidothymidine (AZT) during, through and after delivery, as per the Kenyan guidelines at the time of the study ref [19] if they had attended antenatal clinic

Age at enrollment, nutritional status, residential distance from the hospital and mothers’ HAART status at the time of delivery were independently associated with MTCT of HIV infection (Table 4). Infants enrolled into care after 6 months of age had much higher odds of HIV-infection compared to those enrolled for care within 3 months of age (aOR [95 % CI]: 23.3 [8.3 to 65.4], *p* < 0.001) while infants who exhibited any of the malnutrition syndromes were twice as likely to have acquired HIV-1 (aOR [95 % CI]: 2.3 [1.0 to 5.2], *p* = 0.038). Infants residing more than 10 km from the hospital were twice as likely to acquire HIV infection compared to those living within 5 km of the hospital. Infants born to mothers who were not on HAART at the time of the infant’s birth had more than six-fold odds of HIV infection compared to those born to mothers who had been on HAART for more than 24 months prior to delivery (aOR [95 % CI] 6.5 [1.4 to 29.4], *p* = 0.004).

## Discussion

We report a temporal increase in the proportion of infants enrolling for PMTCT care before 3 months of age and a significant reduction in the proportion of infants enrolled who became HIV-infected, emphasizing the benefits of PMTCT. More than half of the infants completed the 18 months follow-up period, increase from 37 % previously reported at the clinic [20]. Of concern is the 25 % of infants enrolled for care after 3 months of age and therefore less likely to optimally benefit from the care programme. Late presentation for PMTCT care has similarly been reported in an urban setting in Kenya with pregnant mothers making less than four antenatal visits [24]. Such mothers are also more likely to drop out of care [25, 26].

Late enrollment may be an indicator of underlying multifaceted problems [27, 28] or the reflection of a mother only bringing her infant to clinic once the infant is not “thriving”, and they may not have abided to earlier PMTCT requirements [29]. Such mothers would therefore require additional support besides starting PMTCT care. We observed a temporal reduction in overall PMTCT enrollment, a pattern replicated in the number of adults enrolling for HIV care at the clinic, over the same period of time. From 2006, the Kenya government started decentralizing HIV care services from referral hospitals to peripheral clinics. It is likely that access to peripheral care facilities explains the temporal reduction in enrollment since only a minimal reduction in the HIV incidence rate (0.58 % per annum in 2009 to 0.52 % per annum in 2011) has been reported in women (ages 15–49 years) [5].

Once enrolled, 39 % of infants did not complete PMTCT care. We were not in a position to ascertain whether some of those lost to follow up were “self-transfers” to other satellite clinics or true losses from the health care system. Considerable heterogeneity in loss-to-follow up has been reported in sub-Saharan Africa [9, 30]. The recent integration of PMTCT with the MCH service in our setting may help improve retention [31, 32]. Interestingly, a recent report showed no improvement on postnatal service uptake or retention of mothers in care upon integration, however maternal HIV care, enrollment and the use of HAART improved [26]. Integration of PMTCT will also lead to an increased service demand and systems burden at the MCH and this will call for deliberate efforts to ensure that quality care for both PMTCT and MCH services are maintained [33]. The incorporation of expert clients at the facilities, to assist with HAART clinic tasks including measurement of vital signs, anthropometry and counseling, may ease the burden on the health system and improve PMTCT retention [34].

Using the available data we considered potential risk factors for non-retention and their impact on the likelihood of HIV-1 transmission. Previously maternal HIV infection has been associated with poorer WHO growth standards in HIV-exposed uninfected infants followed to 5 years [35] and underweight infants at 1 year [36]. Our observations concur with previous studies that reported sick or malnourished infants to be at higher risk of drop out from care [27, 37]. Higher mortality rates are reported in malnourished children [38–40] and potentially, increased disease and death in this group may partly explain the higher drop out we observed in malnourished infants.

Interestingly, residential distance from the hospital was not associated with retention in care, although other studies have shown that transport costs increase attrition [41]. Choice of a PMTCT facility away from one’s residential area may be a reflection of underlying stigma or health provider trust, although we did not measure this.

Although active follow-up was not randomized, the evidence from the subset of infants actively followed up suggests that this is a valuable intervention to improve PMTCT, as previously observed [42, 43]. Research participation may have improved the mother’s interaction with healthcare providers, understanding and hence attitude towards PMTCT care. Active follow-up poses additional costs, but benefits may outweigh these relative to other interventions against HIV. However, loss-to-follow up has previously been reported at 20 % of HIV-exposed and 14 % of HIV infected infants in spite of an active outreach follow-up and food distribution programme, suggesting that other factors may play a role [37]. Additional interventions may be necessary, such as addressing stigma, improving attitudes towards care programmes for instance through integrated family approaches to improve retention [44], the use of community support groups [45–48] and improving mothers’ economic power [49].

Independently, maternal well-being resulted in better retention, contrary to some observations that maternal well-being was a risk factor for poor retention [25, 28]. Our results agree with previous findings that mothers already receiving HAART were less likely to drop out [30].

We further addressed the impact of the above factors on HIV transmission. We did not include prevalence in 2013, since integration of PMTCT into the MCH altered the programme strategy. Considerable progress has been made in reducing MTCT in the past 7 years. In 2009, the clinic improved its data capture system, which may explain the increased prevalence observed that year. Increased risk of HIV transmission by mothers not on life-long HAART prior to delivery supports the need to improve maternal health for better PMTCT outcomes [7, 50]. Mothers initiating HAART prior to conception have recently been shown to have a lower chance of perinatally infecting their infants, compared to those initiating HAART during pregnancy, in spite of both controlling viraemia [51]. Life-time HAART for the mother may help reduce transmission, improve retention in care and by extension tracking her infant [44, 52, 53]. Late enrollment into care was associated with increased transmission as previously reported [54], such mothers may benefit from timely initiation of HAART. Maternal CD4 count did not correlate with HIV-1 transmission, stratification of CD4 counts with HAART use may have been more informative, but the small sample size hindered a subgroup analysis.

## Conclusion

In conclusion, our results show reducing MTCT over the years and emphasize the benefits of early enrollment into care, adequate infant nutrition and mother’s health for PMTCT success. Observations from our clinic concur with previous reports that the use and retention of mother-infant pairs in PMTCT services is still challenging [27, 37, 44, 55, 56]. Further understanding of factors both cultural and economic that contribute to defaulting PMTCT and the implementation of effective interventions to track and retain infants in PMTCT are needed. In addition measures to reduce missed opportunities from non-enrollment for PMTCT, such as establishing community based PMTCT [57] and participation of traditional birth attendants [58] should be considered. Our results suggest that a simple set of predictors can identify mother-infant pairs at risk of infection or dropout during enrollment in order to implement active intervention to retain them in care.

## Abbreviations

MTCT: Mother to child transmission
PMTCT: Prevention of mother to child transmission
HIV: Human immunodeficiency virus
CCRC: Comprehensive Care and Research Clinic
KCH: Kilifi County Hospital
HAART: Highly active antiretroviral therapy
BMI: Body mass index
WHZ: Weight-for-height z-score
HAZ: Height-for-age Z score
WAZ: Weight-for-age Z score
PCR: Polymerase chain reaction
IQR: Interquartile range
OR: Odds ratios
aOR: Adjusted Odds ratios
CI: Confidence intervals
LRT: Likelihood Ratio Test
HR: Hazard Ratios
pmo: Person months of observation
MCH: Maternal Health Care
